# Changes in the Histology of Walnut (*Juglans regia* L.) Infected with *Phomopsis capsici* and Transcriptome and Metabolome Analysis

**DOI:** 10.3390/ijms24054879

**Published:** 2023-03-02

**Authors:** Leming Zhou, Tianhui Zhu, Shan Han, Shujiang Li, Yinggao Liu, Tiantian Lin, Tianmin Qiao

**Affiliations:** College of Forestry, Sichuan Agricultural University, Chengdu 611130, China

**Keywords:** *Phomopsis capsici*, transcriptome, metabolome, walnut, paraffin section

## Abstract

*Phomopsis capsici* (*P. capsici*) causes branch blight of walnuts, which leads to significant economic loss. The molecular mechanism behind the response of walnuts remains unknown. Paraffin sectioning and transcriptome and metabolome analyses were performed to explore the changes in tissue structure, gene expression, and metabolic processes in walnut after infection with *P. capsici*. We found that *P. capsici* caused serious damage to xylem vessels during the infestation of walnut branches, destroying the structure and function of the vessels and creating obstacles to the transport of nutrients and water to the branches. The transcriptome results showed that differentially expressed genes (DEGs) were mainly annotated in carbon metabolism and ribosomes. Further metabolome analyses verified the specific induction of carbohydrate and amino acid biosynthesis by *P. capsici*. Finally, association analysis was performed for DEGs and differentially expressed metabolites (DEMs), which focused on the synthesis and metabolic pathways of amino acids, carbon metabolism, and secondary metabolites and cofactors. Three significant metabolites were identified: succinic semialdehyde acid, fumaric acid, and phosphoenolpyruvic acid. In conclusion, this study provides data reference on the pathogenesis of walnut branch blight and direction for breeding walnut to enhance its disease resistance.

## 1. Introduction

Walnuts are a quality food for human health and an essential cash crop with important medicine and health functions [1]. Walnut branch blight occurs worldwide and causes threats to the yield and quality of walnuts, seriously jeopardizing the income of fruit farmers and reducing the economic development of walnut-growing regions [1,2]. Walnut branch blight is a plant disease caused by the presence of one or several fungi that mainly affects branches, particularly new branches that are more sensitive to infection. Walnut trees affected with this disease have a low yield and poor quality [3]. *Phomopsis* (*Diaporthe*) is a group of fungi of importance in planting pathology. Numerous fungi in this genus can infect plants and cause disease, resulting in plant ulcers, leaf blight, branch dieback, leaf spot, fruit rot, root rot, and bark necrosis [4,5]. Walnut branches infected with *P. capsici* were collected from Jiange County, Guangyuan City, Sichuan Province, China, in 2017 for pathogenic isolation and identification, and the results indicated that the pathogenic bacterium was *P. capsici*; this was the first report of *P. capsici* causing walnut branch blight disease in China [6]. However, the pathogen’s pathogenic mechanism for walnuts is not yet clear.

Currently, the main chemical control methods are effective against branch blight, but they are very harmful to the environment [7]. Physical control is beneficial but inefficient and time-consuming, and biological control has yet to be widely available. To effectively control this disease, understanding the pathogenesis of the disease and breeding new varieties that are resistant to the disease are issues that are still to be addressed It has been observed in sections of lentils infected with Fusarium acnes that the mycelium first colonizes the bast and begins to expand into the xylem four days after inoculation. In addition, it has been found that the release of phenolic compounds in the early stages of lentil infection with wilt contributes to the delayed development of wilt; the carboxylated polysaccharides secreted by lentils in the later stages of wilt development cause blockage of xylem vessels and disrupt xylem function [8]. In addition, the resistance of plants to pathogen invasion relies mainly on the thickening of cell walls and the formation of structures, such as papillae, invaginations, wall layers, brown matter, and corking of cortical parenchyma cells [9,10].

Host plants undergo substantial physiological, biochemical, metabolic, and phenotypic changes during pathogen invasion. It is widely known that plant recognition of pathogenic bacteria triggers a hypersensitivity response, usually accompanied by programmed cell death in the host plant; the hypersensitivity response includes Ca^2+^ influx, oxidative bursts, and phytohormone signaling [11,12]. On the one hand, the basic ways in which plants respond to pathogen invasion are primarily through the regulation of metabolism-related pathways, including the biosynthesis of secondary metabolites, phenylpropanoid biosynthesis, amino acid and sugar metabolism, photosynthesis, and, to a lesser extent, the regulation of plant–pathogen interactions and MAPK signaling pathways, and the regulation of phytohormones [7]. Plants infected with pathogens are first affected by substances related to the signaling pathway, such as salicylic acid, ethylene, methyl jasmonic acid, methyl salicylic acid, and nitrogen oxides [13,14]; this is followed by primary metabolites, including carbohydrates, organic acids, amino acids, and lipids, and, finally, secondary metabolites, including terpenoids, polyphenols, and mustard oleo sides, which are closely related to plant disease resistance [15,16]. In existing studies, the presence of endophytic *Diaporthe* fungus significantly influenced the metabolic pathways of plants, and the biosynthesis of primary metabolites, such as threonine, malate, and N-acetylmannosamine, which are the precursors of specific metabolites and participate in plant self-defense, was improved [17]. On the other hand, plants may produce toxic substances to kill pathogens or inhibit their growth. It has been shown that the phenolic and organic acid content of different varieties of pepper increases when subjected to *Halyomorpha halys* [18]. Host–pathogen interactions often affect metabolic levels and metabolic pathways in plants [19]. Zeiss et al. identified secondary metabolites involved in plant defense in four tomato species infected with *Ralstonia solanacearum*, with the phenol–propane pathway, which is epitomized by flavonoids and hydroxycinnamic acid, taking an essential role in the defense response [20]. Fungal infection in plants resulted in significant differences in the production of various metabolic components, such as flavonoid compounds, phenols, terpenoids, and various defense proteins, which helped to elucidate the molecular mechanisms of forest tree defense processes and fungal pathogenesis [21,22,23,24].

This study investigated the response of walnuts to *P. capsici* through histological observation of walnut bast and xylem cells and transcriptome and metabolome analyses. The aim is to provide a theoretical basis for the pathogenesis of walnut branch blight and walnut breeding.

## 2. Results

### 2.1. Section Observation of Walnut in Response to P. capsici Infection

**Figure 1:**
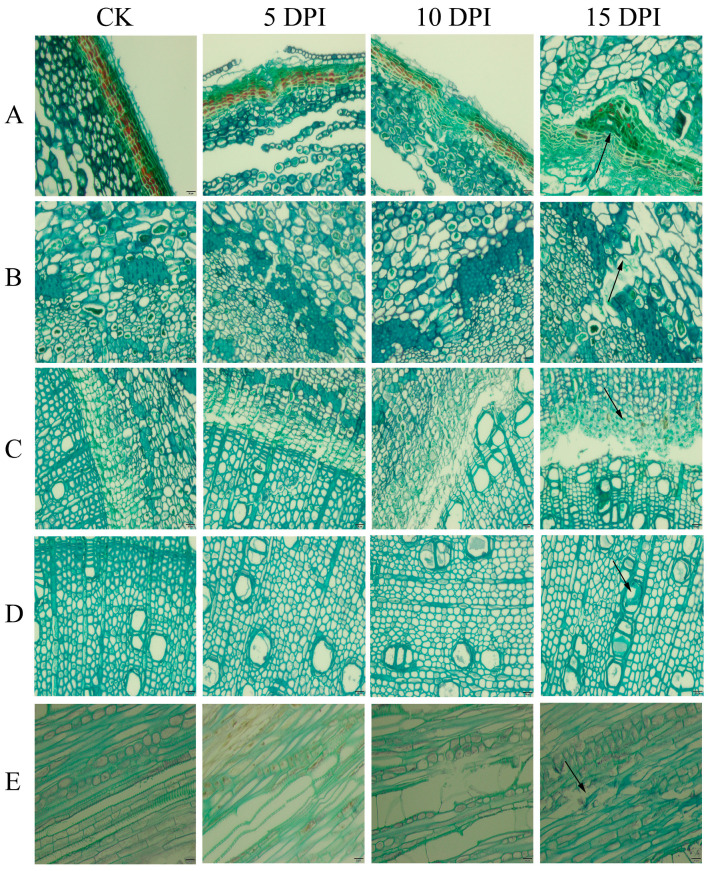
Paraffin sections of uninfected CK and 5 DPI, 10 DPI, 15 DPI walnut branches. The scale bar in the figure is 20 μm. Note: (**A**): periderm; (**B**): phloem; (**C**): cambium; (**D**): xylem cross section; and (**E**): xylem longitudinal section.

Tissue sections of walnut branches are shown in Figure 1. The periderm of walnuts in the control group (CK) group had obvious collenchyma; within these tissues were different-sized and thin-walled parenchyma cells arranged in a close and orderly manner. The interior of the phloem had darkly stained bast fibers. The cambium cells had a clear morphology and were arranged in an orderly manner. The xylem had a large caliber vessel and a clear and clean morphology with neat cell arrangements and structural integrity.

At five days post-infection (5 DPI), the periderm had been slightly invaded, a few parenchyma cells were sparsely arranged, and there were cavities between cells; some xylem cells were broken, and the vessel contained a small amount of dissolved tissue fragment. At ten days post-infection (10 DPI), the periderm showed an obvious invagination: phloem cells were sparsely arranged; cambium cells were lysed; dissolved tissue fragments were visible in the transverse section of the xylem, and a large number of vessel elements were interrupted in the longitudinal section. At fifteen days post-infection (15 DPI), all cells were scattered, and their structures were destroyed; the infection in the vessel could be clearly seen in the transverse section of the xylem.

In summary, after *P. capsici* infection, the internal cell arrangement of walnut branches became gradually more sparse and the structure was destroyed, with the most obvious damage to the xylem.

### 2.2. Transcriptomic Results of Walnut Response to P. capsici Infection

#### 2.2.1. RNA Quality Detection and Sequencing Results

In a total RNA assay, six extracted RNA samples were free of DNA and impurity contamination and degradation, meeting the requirements for library construction. Thus, six cDNA libraries were constructed, and 272,661,616 raw reads were obtained after RNA sequencing. In all, 262,331,938 clean reads (95.82% of the raw reads) were obtained after removing adapter sequences, reads containing poly n, and low-quality reads. Then, the high-quality cleaned read sequences were compared to the reference sequences, and 243,883,980 reads (92.97%) were localized to the reference genome of walnut. The average figures for data Q20 and Q30 were 98.01% and 94.21%, respectively. The GC content was greater than 45.22%. The length of the reads for the six samples was 150 bp. The transcriptome sequencing results met the quality requirements for subsequent assembly analysis. See Table 1 for sequencing information.

#### 2.2.2. Identification of Infected DEG and Functional Analysis

**Figure 2:**
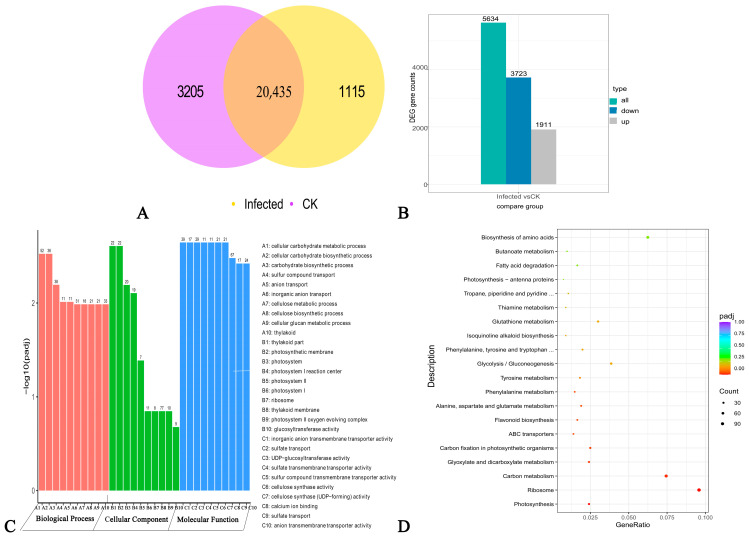
Results of differentially expressed gene analysis. Note: (**A**) DEG Venn diagram; (**B**) DEG volcano map; (**C**) GO enrichment analysis histogram; and (**D**) KEGG enrichment scatter plot.

The results of the DEG analysis for Infected vs. CK combinations are shown in Figure 2. The Infected and CK combinations have 20,435 identical DEGs (Figure 2A). Among these, 5634 genes show significant differences, including 1911 upregulated genes and 3723 downregulated ones (Figure 2B and Table S1). These results indicate that the gene expression of walnut is inhibited by *P. capsici* invasion.

The Gene Ontology (GO) functional enrichment analysis revealed that 1624 DEG are significantly enriched in 52 GO categories, involving 20 biological processes, 5 cellular compositions, and 27 molecular functions (Table S2). The biological processes are mainly “carbohydrate biosynthetic”, “cellulose metabolic”, “cellulose biosynthetic”, “glucan metabolic”, “defense response”, “polysaccharide biosynthetic”, and “glucan biosynthetic processes.” The cell compositions are mainly enriched in “thylakoid”, “thylakoid part”, “photosynthetic membrane”, “photosystem”, and “photosystem I reaction center.” The molecular functions are mainly “glucosyltransferase activity”, “cellulose synthase activity”, “calcium ion binding”, “serine-type exopeptidase activity”, “serine hydrolase activity”, and “structural constituents of ribosomes.” From these results, the 10 most significant terms were selected for each function, and 30 terms were plotted (Figure 2C). There are more downregulated genes than upregulated ones in each main category; however, among the cellular components, ribosomes have more upregulated than downregulated genes (Figure S1). The GO analysis showed that the metabolic processes of cellular carbohydrates are the most significant.

The Kyoto encyclopedia of genes and genomes (KEGG) enrichment analysis showed that 1930 DEGs are annotated to 121 metabolic pathways, among which 367 DEGs are significantly enriched in “photosynthesis”, “ribosomes”, “carbon metabolism”, “glyoxylate and dicarboxylate metabolism”, “carbon fixation in photosynthetic organisms”, “ABC transporters”, “flavonoid biosynthesis”, “alanine”, “aspartate and glutamate metabolism”, and “phenylalanine metabolism” (Table S3). The 20 most significant pathways were selected to draw the scatter plots (Figure 2D). Of these, 88 DEGs are annotated to the carbon metabolism pathway and 113 DEGs are annotated to the ribosome. The KEGG results suggest that carbohydrate metabolism and ribosome-related genes play essential roles in walnut branch blight.

#### 2.2.3. Verification by Real-Time Quantitative Polymerase Chain Reaction (RT-qPCR)

**Figure 3:**
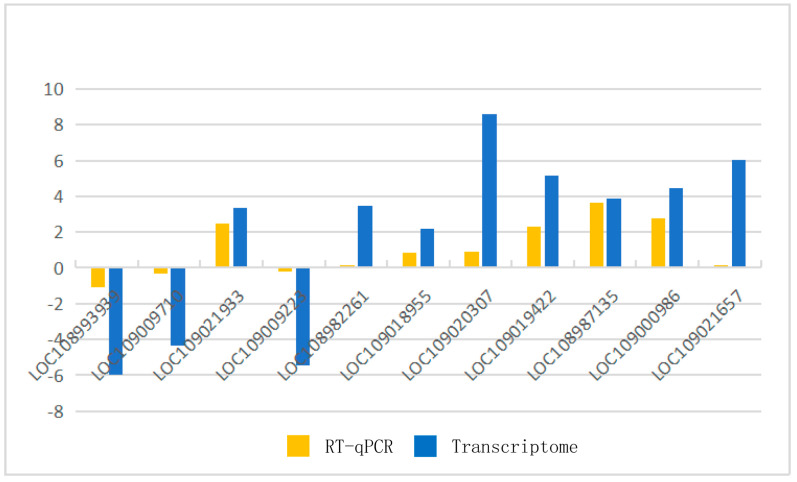
Differences in gene expression were verified by RT-qPCR.

To verify the RNA sequencing data, we used RT-qPCR to assess the expression of walnut genes before and after infection. We randomly selected 11 DEGs to confirm the changes in expression. All genes show the same change trend as the transcriptome sequencing results (Figure 3), proving the reliability of the transcriptome data.

### 2.3. Metabolome Data Analysis

#### 2.3.1. Data Quality Control

**Figure 4:**
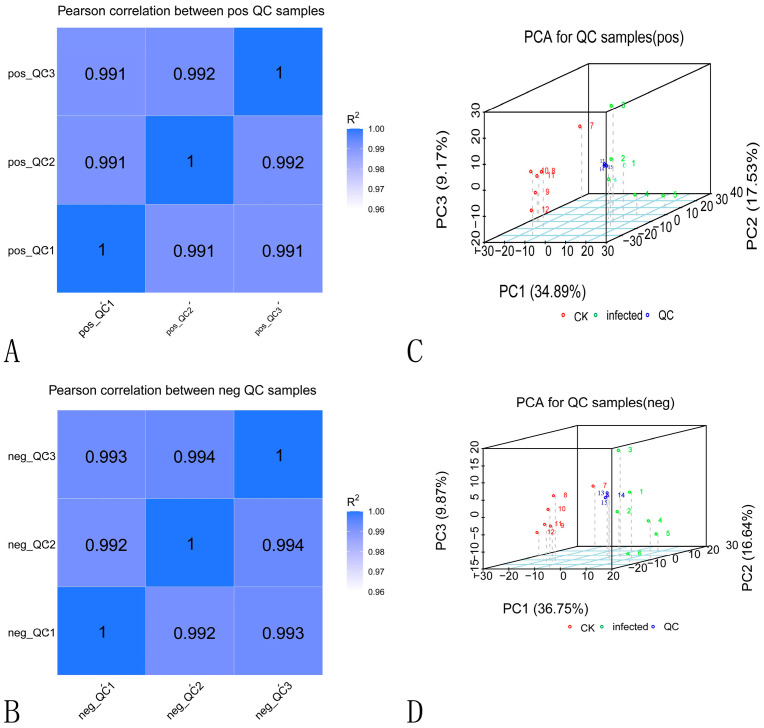
Data quality control chart. Note: (**A**,**B**) QC sample correlation analysis. (**C**,**D**) PCA analysis of QC samples.

Pearson coefficients and principal component analysis (PCA) of the quality control (QC) samples were calculated based on the relative quantitative values of the metabolites; the results are shown in Figure 4. The R2 between the QC samples is close to one (Figure 4A,B), indicating that the whole testing process is constant and the quality of the data are excellent. PCA was used to analyze all QC samples’ pre- and post-infection peaks (Figure 4C,D). The distribution of the QC samples is clustered, indicating that there is minimal variation in the QC samples and the method is stable throughout with the high-quality data.

#### 2.3.2. Metabolite Pathways and Classification Notes

**Figure 5:**
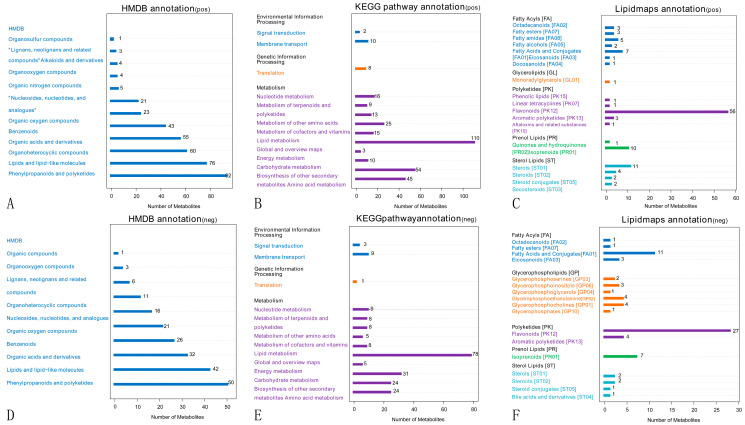
Annotated diagram of metabolite pathway and classification. Note: (**A**,**D**) Annotated HMDB taxonomic map of metabolites. (**B**,**E**) Annotated map of the KEGG pathway of metabolites. (**C**,**F**) Annotated map of LIPID MAPS classification of metabolites.

According to the Human Metabolome Database (HMDB) in the pos (Figure 5A) and neg modes (Figure 5D), 92 and 50 metabolites correspond to phenylpropanoids and polyketones, 76 and 42 metabolites correspond to lipids and lipid-like molecules, and 60 and 32 metabolites correspond to organic heterocyclic compounds. The metabolites identified by the secondary profiles were subjected to the KEGG website for metabolic pathway analysis. In the pos (Figure 5B) and neg modes (Figure 5E), most metabolites were mainly annotated to the global and overview maps, amino acid metabolism, biosynthesis of other secondary metabolites, and carbohydrate metabolism. LIPID MAPS annotation was performed on the identified metabolites, and the results showed that in the pos (Figure 5C) and neg modes (Figure 5F), the metabolites were mainly enriched in flavonoids, fatty acids and conjugates, and isoprenoids. The results of the metabolite annotation are shown in Figure 5.

#### 2.3.3. DEM Screening and KEGG Pathway Annotation

The two groups were screened for differences in metabolites. In total, 993 metabolites were screened under the pos mode, and 434 metabolites were differentially expressed (254 upregulated and 180 downregulated); 521 metabolites were screened under the neg mode, and 226 metabolites were differentially expressed (120 upregulated and 106 downregulated).

**Figure 6:**
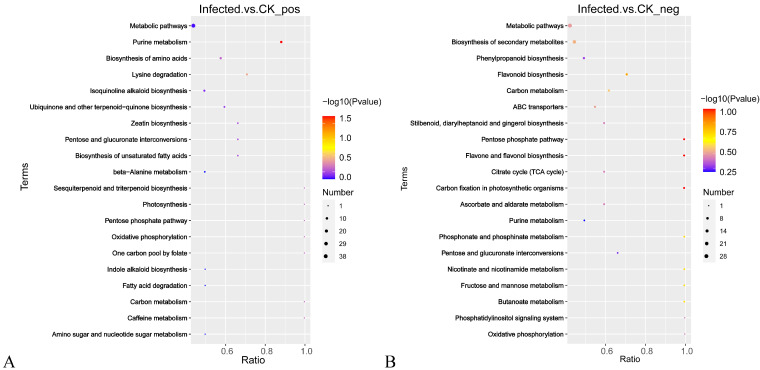
DEM annotation diagram. Note: KEGG pathway classification of DEM in pos (**A**) and neg (**B**).

The metabolites screened for KEGG annotation found that, in the pos mode, a total of 156 metabolites were annotated, of which 69 differential metabolites were annotated to 22 metabolic pathways (Table S4). In the neg mode, 99 metabolites were annotated, of which 39 differential metabolites were annotated to 32 metabolic pathways (Table S5). Based on these results, bubble maps of the top 20 enriched pathways were drawn, as shown in Figure 6. The pentose phosphate pathway, carbon fixation in photosynthetic organisms, and flavone and flavonol biosynthesis are the most enriched. All three differential metabolites (rutin, kaempferol, and luteolin) annotated in flavone and flavonol biosynthesis are reduced in levels. The metabolites D-Xylulose 5-phosphate and D-Sedoheptulose 7-phosphate are jointly annotated in the pentose phosphate pathway and the carbon fixation pathway of photosynthetic organisms and express downward, indicating that these metabolites play an important role in walnut branch blight.

#### 2.3.4. Transcriptome and Metabolome Association Analysis

A comprehensive transcriptome and metabolome analysis was performed using the Pearson correlation coefficient method to identify statistically significant genes and metabolites. The top 50 DEMs and the top 100 DEGs with significant correlation in the pos (Figure S2) and neg (Figure S3) ion modes were identified, and KEGG analysis was performed. The results of the transcriptomic and metabolomic KEGG association analysis found that, in the pos (Table S6) and neg (Table S7) modes, the enrichment categories involved in DEMs and DEGs are membrane transport, signal transduction, amino acid metabolism, biosynthesis of other secondary metabolites, carbohydrate metabolism, energy metabolism, lipid metabolism, metabolism of cofactors and vitamins, metabolism of other amino acids, metabolism of terpenoids and polyketides, and nucleotide metabolism. The main pathways enriched are 10 metabolic pathways involved in the annotation to carbohydrate metabolism, 6 annotations to amino acid metabolism, 4 annotations to the biosynthesis of other secondary metabolites, and 4 annotations to the metabolism of cofactors and vitamins. The results are shown in Table 2.

In carbohydrate metabolism, intermediate metabolites decrease in all pathways, except for d-sorbitol and xylitol, including fumaric acid, phosphoenolpyruvic acid, cis-Aconitic acid, D-Xylulose 5-phosphate, D-Galacturonic acid, etc. The normal metabolism of carbohydrates is an essential basis for plant growth and development. It follows that the invasion of *P. capsici* inhibits walnut development. In amino acid metabolism, metabolites 3-hydroxy phthalic acid, glutathione, fumaric acid, succinic semialdehyde, phenylglyoxal, lecithin, N6-acetyl-L-lysine, 2-oxo adipic acid, and glutaric acid are decreased, while only N-acetyl-L-phenylalanine and L-glycine are increased. In the biosynthesis of other secondary metabolites, the number of up-regulated DEMs is greater than the number of down-regulated DEMs. In nicotinate and nicotinamide metabolism, vitamin B6 metabolism, ubiquinone and other terpenoid–quinone biosynthesis, and one carbon pool by folate pathways, fumaric acid, succinic semialdehyde, succinic semialdehyde, p-coumaric acid, and shikonin and folinic acid are decreased, while only phylloquinone is increased, as shown in Table 2. In this study, it is found that *P. capsici* infestation inhibits the metabolism and synthesis of carbohydrates (pyruvate, butanoate, pentose, glucuronate, fructose, and mannose), amino acids (tyrosine, cysteine, methionine, alanine, aspartate, glutamate, phenylalanine, tryptophan, and lysine), and other secondary metabolites (terpenoids and vitamins) in walnuts. However, the synthesis of secondary metabolites (mainly flavonoids) is enhanced. Remarkably, succinic semialdehyde acid, fumaric acid, and phosphoenolpyruvic acid are annotated to multiple metabolic pathways, indicating that these three metabolites play essential regulatory roles in the metabolism of walnuts.

## 3. Discussion

We used a combination of paraffin tissue sectioning, transcriptome sequencing, and metabolomics to analyze walnut branch blight and provide a theoretical basis for enhancing disease resistance in walnuts.

Like many blight pathogens, *P. capsici* targets xylem vessel molecules [25]. Pathogens enter the epidermis through the wounds, continue through the cortex and endodermis, and eventually reach the xylem, where they proliferate and spread [26]. The disintegration of the cortex and cambium cells primarily affects the division of plant cells and the transport of nutrients [27,28,29]. The primary role of the xylem in plants is to transport water, minerals, and numerous signal molecules [30,31,32,33]; this tissue consists of lignified vessel elements, fibers, and parenchyma cells. It has been suggested that plants of susceptible species have larger vascular molecules, a feature that may benefit infection [34]. The proliferation of xylem vascular wilt pathogens in the xylem leads to a breakdown of water and mineral transport, which leads to heavy wilting and death among infected plants. Remarkably, the xylem is found to block infection through the production of phenolic compounds, which is in line with the results of previous studies [8,35].

At post-infection, the expression of most genes in walnut is suppressed. Among them, the most DEGs are annotated in carbon metabolism and ribosomes. The expression of intermediate metabolites of the pentose phosphate pathway (PPP) and the carbon fixation pathway of photosynthetic organisms was suppressed in walnut. Carbon metabolism is the most important basic metabolism in the life cycle of plants, involving in the degradation and conversion of photosynthetic products, such as starch and sucrose synthesis, as well as respiratory processes, such as glycolysis, the tricarboxylic acid cycle, the pentose phosphate pathway, the ethanoic acid oxidation pathway, and the glyoxalate cycle, which provide the necessary raw materials and energy for amino acid, protein, and nucleic acid synthesis. Carbon metabolic processes in plants are closely related to plant growth and may also be involved in mechanisms of plant disease resistance [36,37,38]. Downregulation of genes related to carbon metabolism affects the levels, distribution, and imbalance of soluble sugars and starch in stem xylem and bast, and the pathways related to sugar and starch metabolism, thereby promoting plant disease development [38]. Ribosome is the site of intracellular protein synthesis and is essential in plant growth, development, and defense responses [39]. Upon infestation by pathogenic bacteria, host plants induce the production and accumulation of various defense-related genes and pathogenesis-related proteins (PR) [40]. Therefore, in this study, we hypothesized that up-regulated expression of ribosomal genes enables host plants to produce more PRs to resist pathogenic attacks.

There are many metabolic functions in which the PPP performs a key role, including the generation of NADPH, biosynthesis of nucleotides, and carbon homeostasis [41,42]; this pathway produces four-, five-, and seven-carbon compounds and transketolases and transaldolases, which are also related to photosynthesis. Many existing studies have shown that the intermediate metabolites of the PPP play a crucial role in plant resistance to pathogenic bacterial attack [43,44,45]; therefore, the PPP is inhibited and the plant’s carbon fixation is slowed down in response, which in turn affects walnut growth and development, and increases plant susceptibility to disease.

Amino acids combine to form proteins and are also the precursors for the synthesis of many plant hormones [46]; some amino acids (e.g., proline) can enhance plant resistance [47,48,49]. Some vitamins are recognized as essential antioxidants and work in stress response [50], such as a weakened biosynthesis of vitamin B6 promotes plant susceptibility to disease [51]. Terpenoids are widespread; some are involved in fundamental plant processes, such as photosynthesis, respiration, and growth and development. Some specialized metabolites show a wide range of biological activities, including antibacterial, anti-disease and anti-inflammatory actions, cytotoxic actions, and anti-tumor agent and enzyme inhibition [52,53].

Phenylpropanoid metabolite biosynthesis is a complex network that produces various critical secondary metabolites, including lignans and naringin, and its regulatory mechanism is essential for plant growth, development, and biotic stress protection. There are two main roles attributed to lignans and naringin: plant defense and antioxidant activity [49,54]. Flavonoids and flavonols in plants have important defensive functions against fungi [50,51]. In plants, flavonols serve to protect plants against various stimuli of the environment. Kaempferol and lignan are common polyphenolic substances, and rutin is the most common flavonol glycoside compound; they have antiviral, antifungal, and anti-biofilm abilities, as well as antioxidant activity and pharmacological properties [53]. In our study, increased walnut phenolic compounds facilitated the resistance to *P. capsici* infestation. Succinic semialdehyde acid is a significant metabolite of gamma-aminobutyric acid. The results of previous studies suggest that succinate semialdehyde functions initiate a quorum-quenching mechanism, which reduces quorum-sensing signals and, thus, avoids eliciting plant defense, Succinic semialdehyde acid gene silencing has been reported to increase disease susceptibility in tomatoes [55]; it has also been shown that succinic acid can enhance the viability of bacteria throughout an infection [56]. Fumaric acid is an organic acid that positively affects resistance to fungi, and plants can inhibit pathogen colonization and survival through the release of fumaric acid [57,58,59]. However, some studies have shown that the accumulation of fumaric acid can cause accelerated decay in plants [60], and it is found in *Panax notoginseng* that fumaric acid stimulates fungal growth and chemotaxis. Fungal antagonistic activity may also be affected by fumaric acid [61]. Rice induces phosphoenolpyruvic acid when being treated with insecticides to defend itself against insecticide damage [61]. Previous studies have also reported that phosphoenolpyruvic acid accumulates under abiotic stresses, such as blue light [62], high salt [63] and low nitrogen concentrations [64], and it is assumed that phosphoenolpyruvic acid can enhance the resistance of organisms to stress. Proper metabolism of organic acids can promote plant growth and disease resistance, but too much or insufficient metabolism can cause plant growth disruptions. In this study, succinic semialdehyde acid, fumaric acid, and phosphoenolpyruvic acid were significantly suppressed in walnut post-infection, confirming that the walnut variety “xiangling” was sensitive to *P. capsici*. In this study, infection of walnuts by *P. capsici* resulted in the gradual lysis of walnut cells; the inhibition of the expression of genes related to the carbon metabolism pathway (mainly PPP); the enhanced expression of genes in the ribosome; and the inhibition of the biosynthesis and metabolism of metabolite pathways, such as carbohydrates, and amino acids and cofactors, while enhancing the biosynthesis of secondary metabolites. We hypothesized that *P. capsici* infection caused a dysregulation of sugar and organic acid production in walnuts, thereby disrupting intracellular homeostasis and leaving walnuts in a susceptible state. At this point, *P. capsici* induced cellulase reactive oxygen species, causing a hypersensitive cellular response that induced programmed cell death and leading to the cell lysis phenomenon presented on walnut sections. At the same time, walnuts responded to the pathogenic stress by increasing the expression of secondary metabolites (e.g., prunin, luteolin, kaempferol, and (−)-epigallocatechin).

In conclusion, this study provides data reference on the pathogenesis of walnut branch blight and direction for breeding walnut to enhance its disease resistance. However, the exact mechanism of how walnut resists *P. capsici* is open to debate.

## 4. Materials and Methods

### 4.1. Plant Materials and Pathogenic Bacterial Inoculation

The walnut variety “xiangling” from Guangyuan City, Sichuan Province, China, was used as the material. The preserved *P. capsici* strain was inoculated in a traditional PDA medium. After 3 days of activation, the cake was punched out with a 5 mm diameter punch for inoculation experiments. The inoculation sites were annual branches of walnut. The inoculation area was disinfected with 75% alcohol, rinsed with sterilized water, and dried. In the middle part of the inoculation zone, a wound of 1 cm × 1 cm size was scraped out with a knife, and the prepared bacteriophage cake was inoculated onto the wound. The branches were inoculated with a sterile medium as a control (CK), and each treatment was set up with nine biological replicates. These treatments were placed in the College of Forestry, Sichuan Agricultural University, until the onset of the disease.

### 4.2. Paraffin Sectioning of Walnut Branches

The branches were infected with *P. capsici* at different times (5 DPI, 10 DPT, and 15 DPI), and CK were selected for sectioning. Conventional paraffin sectioning was used. The stem samples were cut transversely into 5 mm thick segments and placed in a FAA fixative for 24 h. Then, the samples were placed in a dehydration chamber with a plant-softening solution (Wuhan Safeway Biotechnology Co., Ltd., Wuhan, China). The softening fluid treatment occurred for seven days and the fluid was changed daily. After softening, the samples were rinsed with running water for 30 min; soaked in 15% ethanol for 2 h, 30% alcohol for 1 h, 50% alcohol for 1 h, 75% alcohol for 1.5 h, 90% alcohol for 1.5 h, 1.5% alcohol for 5 h, 100% alcohol for 1 h, xylene for 8 min, and xylene for 20 min; and dehydrated after each soaking. The samples were placed in paraffin wax for 30 min, paraffin wax for 1 h, and paraffin wax for 2 h; then, they were placed on an embedding rack and removed and trimmed after the wax solidified. The sections were cut to a thickness of 5 μm, dried, and stored at room temperature. Next, the sections were placed in xylene I for 20 min, xylene II for 20 min, anhydrous ethanol I for 5 min, anhydrous ethanol II for 5 min, and 75% alcohol for 5 min; then, they were washed with tap water. The sections were treated with toluidine blue for 2–5 min, rinsed with tap water, placed in a clean xylene clearing solution (Sinopharm Chemical Reagent Co., Ltd., Shanghai, China) for 10 min, sealed with a neutral glue (Sinopharm Chemical Reagent Co., Ltd.), and finally observed under microscopy.

### 4.3. RNA Sequencing

The 15 DPI was the critical time when the yellow-brown spots were just beginning to appear on the inoculated walnut branches, which was the beginning of the walnut branch blight. A 1 cm × 2 cm section of the bark was taken immediately below the diseased tissue as a sample, and from the exact location in the CK, three replicates of each group were made. The samples were labeled and frozen in liquid nitrogen, and RNA extraction was immediately performed. Total RNA was extracted using the phenol-chloroform method. Sample quality was inspected on an Agilent 2100 Bioanalyzer system by assessing RNA integrity. Transcriptomes were sequenced on an Illumina NovaSeq 6000 (Illumina, San Diego, CA, USA) platform by Novogene Bioinformatics Technology Co. (Beijing, China) The raw RNA-seq data had been submitted to the National Genomics Data Centre. (Bioproject: PRJNA867174).

### 4.4. Transcriptome Analysis

The clean reads were obtained by fine filtering after RNA sequencing to remove linker information, low-quality bases, and non-detected bases from the raw reads. Q20, Q30, and GC content were calculated for the clean reads, and the clean reads were compared to the reference genome using the HISAT2 software. DEG was identified using fragment per million exons mapping (FPKM), and genes with absolute values |log2(Fold Change)| ≥ 1 & padj ≤ 0.05 were considered significant DEGs. DEGs were annotated according to the non-redundant database (Nr), SwissProt/UniProt Plant Protein, KEGG, and eggNOG. Then, they were analyzed for enrichment of the KEGG pathways and GO functions.

### 4.5. RT-qPCR Verification

To further confirm the expression levels of genes, RT-qPCR analyses were performed. We used cDNAs of mRNA reverse transcripts from the sequenced samples as templates and randomly selected 11 genes with high differential multiplicity, including upregulated DEGs and downregulated DEGs, with GAPDH as a reference gene. The RT-qPCR primers were designed using the CDS regions of 11 candidate genes, with GAPDH as the internal reference gene, and the Primer Premier 5.0 software was used for primer design (Supplementary Table S8). The primers were 18–25 bp in length, and the target amplification bands were 150–300 bp. Changes in the expression of the 11 candidate DEGs were quantified using a fluorescent quantitative polymerase chain reaction (CFX96-Real-Time System, San Diego, CA, USA). The RT-qPCR system was composed of the following parts: 10 μL Mix (Servicebio, Chengdu, China), 8 μL ddH2O, F/R 0.5 μL, and 1 μL cDNA (CK/Infected). The RT-qPCR was carried out as follows: 94 °C for 20 s, 94 °C for 10 s, and 60 °C for 20 s, and 38 cycles were repeated from step 2 to step 3. Three replicates of each RT-qPCR run were performed, the mean values were calculated, and the data were analyzed using the 2^–△△^Ct method.

### 4.6. Metabolite Extraction and Detection

The samples were taken in the same manner and at the same sites as for the transcription group, with six sample replicates. Then, 100 mg of walnut tissues were taken from each sample, ground in liquid nitrogen, and placed separately in EP tubes. The sample was extracted as follows: 500 µL aqueous 80% methanol was added to the vortex suspension homogenate; the supernatant was incubated on ice for 5 min, centrifuged at 15,000× *g* for 20 min at 4 °C, and diluted with LC-MS-grade water to a concentration containing 53% methanol; and then the supernatant was centrifuged again at 15,000× *g* for 20 min at 4 °C and injected into the LC-MS/MS system for analysis. Using a Vanquish UHPLC system (Thermo Fisher, Berlin, Germany) and an Orbitrap Q Exactive^TM^HF-X mass spectrometer (Thermo Fisher, Germany), we carried out UHPLC-MS/MS analysis at Novogene Ltd. (Beijing, China). The testing parameters were default company standard parameters.

### 4.7. Data Processing and Metabolite Identification

The (raw) files were processed using the CD 3.1 library search software, setting a quality deviation of 5 ppm, a signal intensity deviation of 30%, a signal-to-noise ratio of 3, a minimum signal intensity, and summing the ions. Simultaneously, the peak areas were quantified, and then the target ions were integrated; this was followed by molecular ion peaks and fragment ions for molecular formula prediction and comparison with the mzCloud, mzVault, and Masslist databases. The background ions were removed using the blank samples, and the raw quantification results were normalized.

### 4.8. Data Analysis

The KEGG database (https://www.genome.jp/kegg/pathway.html, accessed on 13 February 2022), the HMDB database (https://hmdb.ca/metabolites, accessed on 14 February 2022), and the LIPIDMaps database (https://www.lipidmaps.org/, accessed on 16 February 2022) were used to annotate the metabolites. PCA and partial least squares discriminant analyses were performed using metaX, and the VIP values were available for each metabolite. In the univariate analysis, statistical significance (*p*-value) and fold change (FC) were calculated for the metabolites based on *t*-tests. The default criteria for DEM screening are VIP > 1, *p* value < 0.05, and FC ≥ 2 or FC ≤ 0.5. The volcano map was plotted using the R package ggplot2; the clustering heat map was created using the R package Pheatmap; the metabolite data were normalized using z-score; the Pearson correlation coefficient was performed using the R language cor (); statistical significance was achieved using cor.mtest() in R; the correlation map was plotted using the R language complot package; and the KEGG database was used to study metabolite function and metabolic pathways.

### 4.9. Metabolome and Transcriptome Association Analysis

We selected CK1, CK2, CK3, Infected 1, Infected 2, and Infected 3 of the transcription group for quantitative comparative analysis with CK1, CK2, CK3, Infected 1, Infected 2, and Infected 3 of the metabolism group. Pearson correlation analysis and KEGG enrichment analysis were performed for the CK vs. Infected group, and the results were screened according to the criteria of *p* < 0.05. Metabolic-transcriptional KEGG enrichment bubble maps were plotted using the ggplot2 package in R language for the co-enrichment pathways.

## Figures and Tables

**Table 1:** Sequencing data for the six samples.

Sample	Raw Reads	Clean Reads	Error Rate	Q20 (%)	Q30 (%)	GC pct (%)	Total Map
Infected1	45,456,664	44,189,378	0.02	98.16	94.49	46.33	38,911,491 (88.06%)
Infected2	46,940,018	45,063,852	0.03	97.78	93.85	46.03	42,362,147 (94.0%)
Infected3	45,204,774	43,300,026	0.02	98.07	94.30	46.45	38,563,666 (89.06%)
CK1	45,829,520	43,916,056	0.02	98.11	94.36	45.55	41,962,692 (95.55%)
CK2	46,463,026	44,739,028	0.02	98.12	94.31	45.22	42,973,409 (96.05%)
CK3	42,767,614	41,123,598	0.03	97.83	93.93	45.55	39,110,575 (95.1%)

**Table 2:** Metabolomic and transcriptomic co-enrichment results in pathway entries and expression of DEGs and DEMs.

Category	Pathways	DEM Down	DEM Up
Carbohydrate metabolism	Pyruvate metabolism	Fumaric acid; Phosphoenolpyruvic acid	
	C5-branched dibasic acid metabolism	Itaconic acid; cis-Aconitic acid	
	Ascorbate and aldarate metabolism	Ascorbic acid; D-Xylulose 5-phosphate; D-Glucarate	
	Fructose and mannose metabolism	α-D-Mannose 1-phosphate	D-Sorbitol
	Pentose phosphate pathway	D-Gluconic acid; D-Xylulose 5-phosphate; D-Sedoheptulose 7-phosphate; Gluconolactone	
	Butanoate metabolism	Fumaric acid; Succinic semialdehyde	
	Inositol phosphate metabolism	D-myo-Inositol 1,4-bisphosphate	
	Citrate cycle (TCA cycle)	Fumaric acid; cis-Aconitic acid; Phosphoenolpyruvic acid	
	Pentose and glucuronate interconversions	D-Galacturonic acid; D-Xylulose; D-Xylulose 5-phosphate;	Xylitol
	Amino sugar and nucleotide sugar metabolism	D-Galacturonic acid	
Amino acid metabolism	Tyrosine metabolism	Fumaric acid; Succinic semialdehyde	
	Cysteine and methionine metabolism	Glutathione	
	Alanine, aspartate, and glutamate metabolism	Fumaric acid; Succinic semialdehyde	
	Phenylalanine metabolism	Fumaric acid; N-Acetyl-L-phenylalanine;	Phenylacetaldehyde
	Tryptophan metabolism	3-Hydroxyanthranilic acid	
	Lysine degradation	L-Saccharopine; 2-Oxoadipic acid; Glutaric acid	N6-Acetyl-L-lysine; Pipecolic acid;
Biosynthesis of other secondary metabolites	Phenylpropanoid biosynthesis	Scopoletin; Scopolin; Chlorogenic acid	Sinapyl Alcohol; Cinnamaldehyde;
	Flavonoid biosynthesis	Chlorogenic acid;	Neohesperidin; (−)-Epigallocatechin; Pinocembrin; Prunin; Taxifolin; Naringin; Dihydromyricetin; 5,7,3′,4′,5′-Pentahydroxyflavone; Kaempferol; Luteolin
	Stilbenoid, diarylheptanoid, and gingerol biosynthesis	Bisdemethoxycurcumin; Chlorogenic acid;	Resveratrol
	Isoquinoline alkaloid biosynthesis	Tyramine; p-Coumaric acid	Corydaline; 3,4-Dihydroxybenzaldehyde; Lycorine
Metabolism of cofactors and vitamins	Nicotinate and nicotinamide metabolism	Fumaric acid; Succinic semialdehyde	
	Vitamin B6 metabolism	Succinic semialdehyde	
	Ubiquinone and other terpenoid–quinone biosynthesis	p-Coumaric acid; Shikonin	Phylloquinone
	One carbon pool by folate	Folinic acid	

## Supplementary Materials

The following supporting information can be downloaded at: https://www.mdpi.com/article/10.3390/ijms24054879/s1.
