# Supplementary figures and images for: Changes in the Histology of Walnut (Juglans regia L.) Infected with Phomopsis capsici and Transcriptome and Metabolome Analysis
Source: Int J Mol Sci. 2023 Mar 2;24(5):4879. doi: 10.3390/ijms24054879 (PMC10003368; doi:10.3390/ijms24054879)

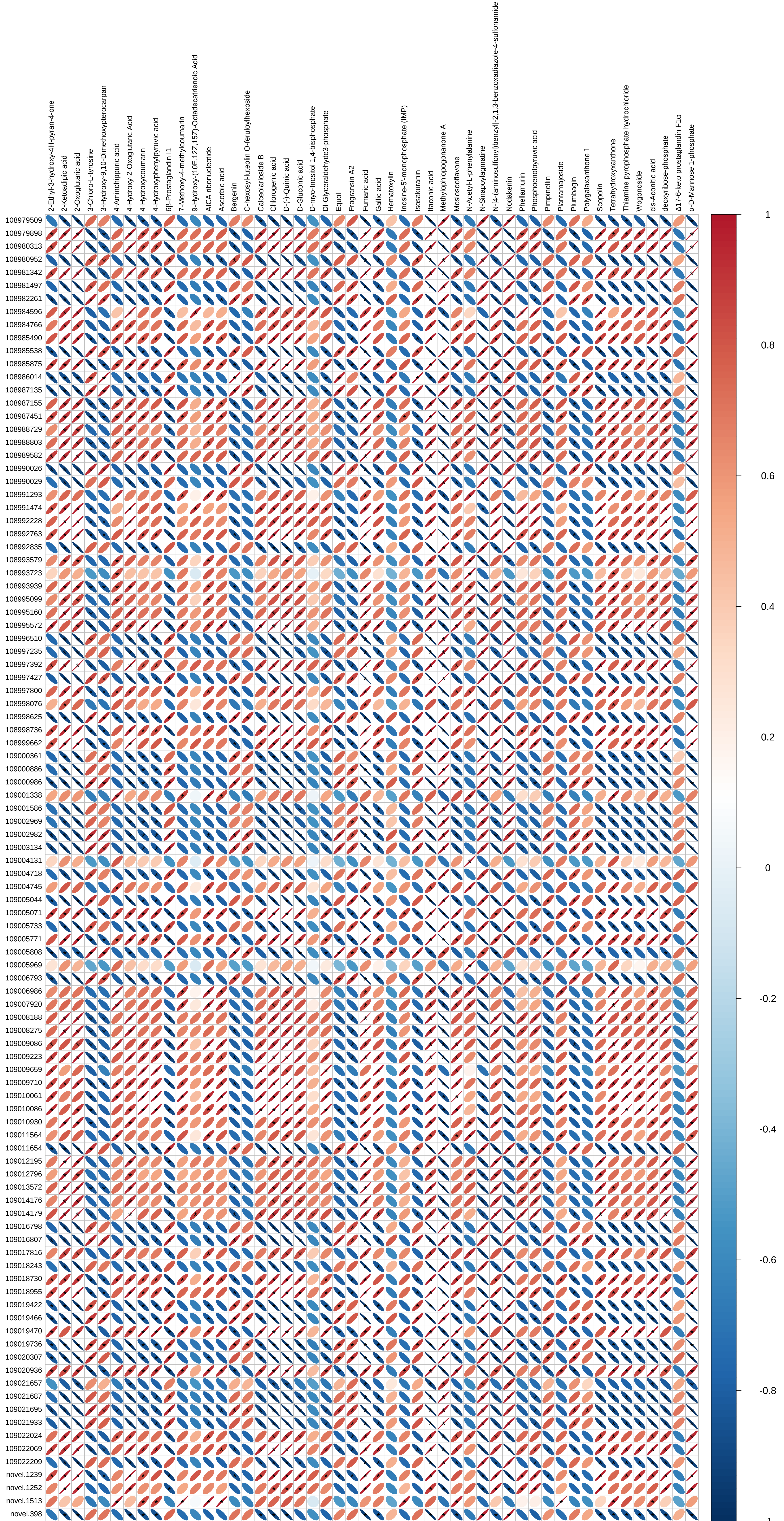

Supplement: Supplementary file 1 [file ijms-24-04879-s001.zip › Figure S3.pdf]

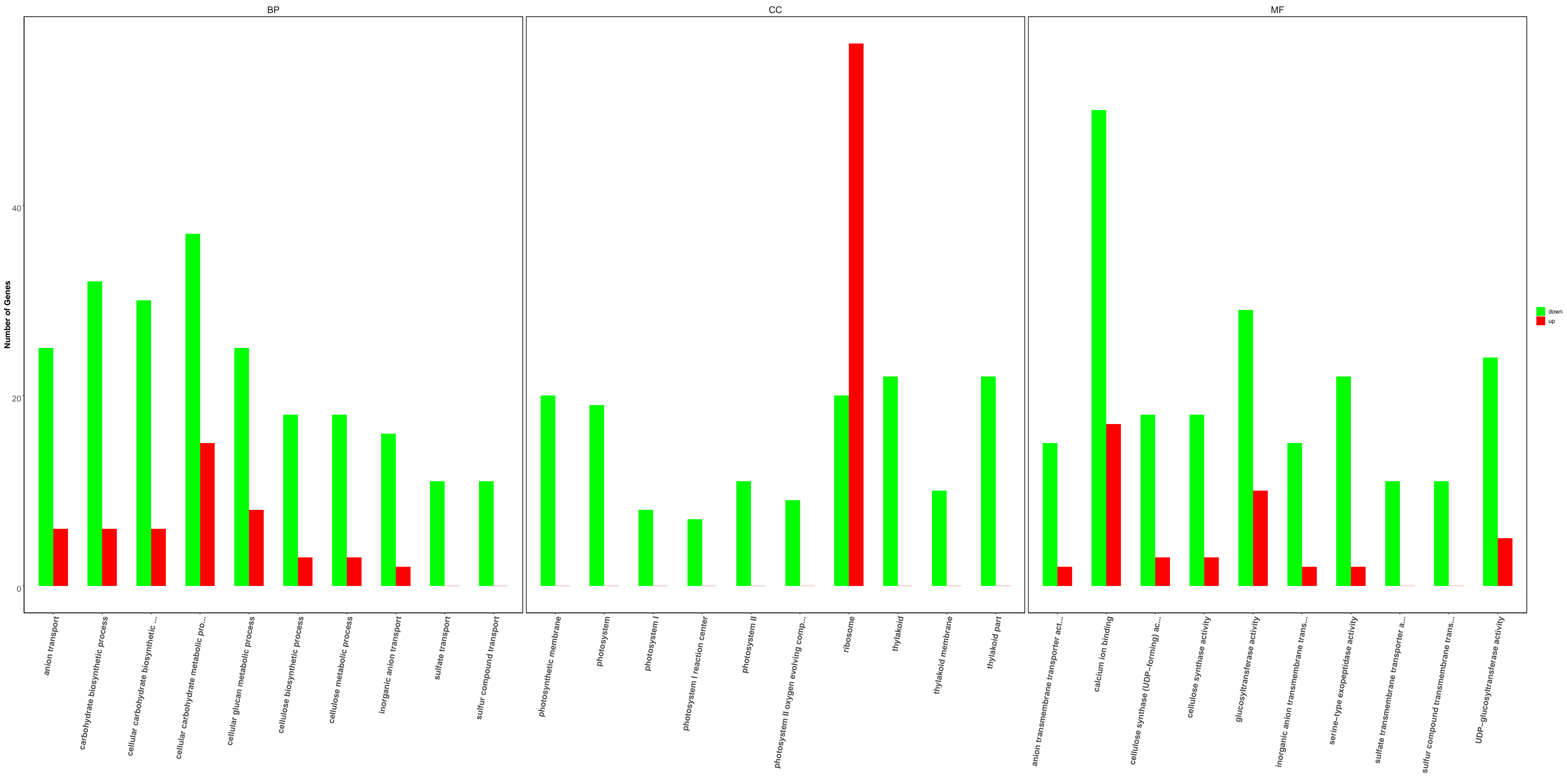

Supplement: Supplementary file 1 [file ijms-24-04879-s001.zip › Figure S1.pdf]

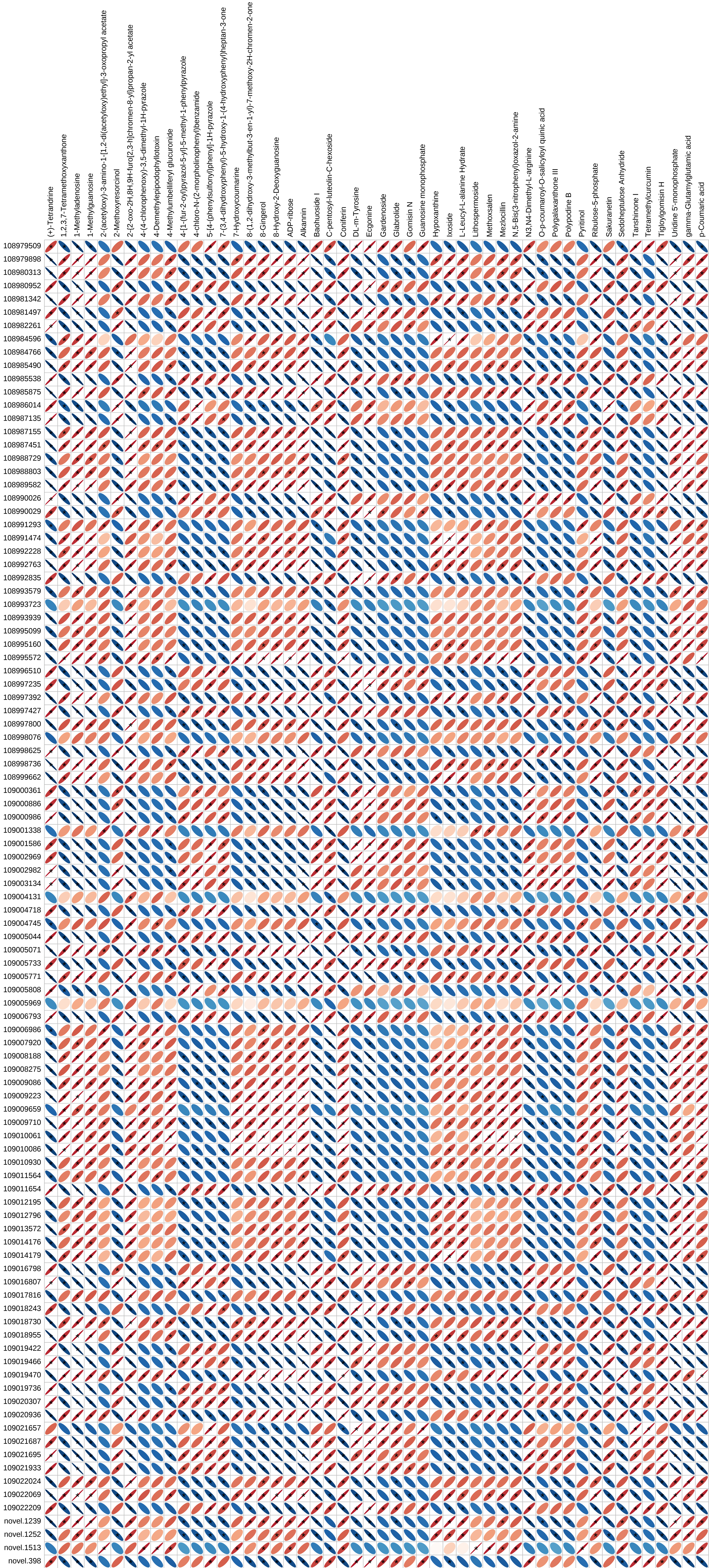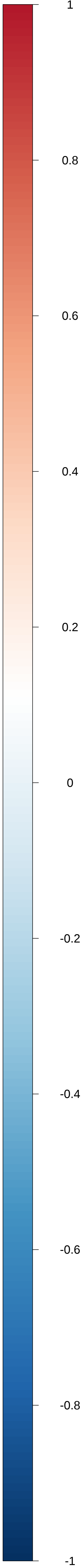

Supplement: Supplementary file 1 [file ijms-24-04879-s001.zip › Figure S2.pdf]
